# Evaluation of safety and efficacy of gefitinib ('iressa', zd1839) as monotherapy in a series of Chinese patients with advanced non-small-cell lung cancer: experience from a compassionate-use programme

**DOI:** 10.1186/1471-2407-4-51

**Published:** 2004-08-19

**Authors:** Xin-Lin Mu, Long-Yun Li, Xiao-Tong Zhang, Shu-Lan Wang, Meng-Zhao Wang

**Affiliations:** 1 Department of Respiratory Diseases, Peking Union Medical College Hospital, Peking Union Medical College and Chinese Academy of Medical Sciences, Beijing 100730, China

## Abstract

**Background:**

The gefitinib compassionate-use programme has enabled >39,000 patients worldwide to receive gefitinib ('Iressa', ZD1839) treatment. This paper reports the outcome of gefitinib treatment in Chinese patients who enrolled into the 'Iressa' Expanded Access Programme (EAP) at the Peking Union Medical College Hospital.

**Methods:**

Thirty-one patients with advanced or metastatic non-small-cell lung cancer (NSCLC) that had progressed after prior systemic chemotherapy were eligible to receive oral gefitinib 250 mg/day as part of the EAP. Treatment was continued until disease progression or unacceptable toxicity occurred. The impact of treatment on disease-related symptoms and quality of life (QoL) was evaluated with the Chinese versions of European Organization for Research and Treatment of Cancer Quality of Life Questionnaires (EORTC QLQ-C30 and QLQ-LC13).

**Results:**

Gefitinib was well tolerated. Adverse events (AEs) were generally mild (grade1 and 2) and reversible. The most frequent AEs were acneform rash and diarrhoea. Only one patient withdrew from the study due to a drug-related AE. The objective tumour response rate was 35.5% (95% confidence interval [CI]: 18.6–52.3); median progression-free survival was 5.5 months (95% CI, 1.6 to 9.4); median overall survival was 11.5 months (95% CI, 5.6 to 17.3). The QoL response rates for five functioning scales and global QoL varied from 56–88%. The main symptom response rates varied from 44–84%. QoL and symptom response were correlated with objective tumour response.

**Conclusion:**

Gefitinib demonstrated safety and efficacy as monotherapy in this series of Chinese patients with advanced NSCLC and was also associated with remarkable symptom relief and improvement in QoL. Although clinical trials are needed to confirm these positive findings, the data suggest that treatment with gefitinib may be beneficial for some Chinese patients who do not respond to chemotherapy and have poor prognosis.

## Background

Platinum-based combination chemotherapy is the standard first-line treatment for patients with advanced non-small-cell lung cancer (NSCLC). Although meta-analysis of clinical trials proved that combination chemotherapy for advanced NSCLC is superior to best supportive care [1] and new cytotoxic agents have been developed over the past decade, the 5-year survival rate for these patients remains <1% [2]. The pace of progress is too slow and new therapeutic approaches are required. Based on our increasing understanding of the biology of NSCLC, targeted therapy is providing some promising new agents. Targeted therapy is usually aimed at a key protein implicated in tumour cell proliferation, survival, invasion or resistance to conventional treatments but spares the normal cells, thereby producing less toxicity than conventional therapies [3].

Many solid tumours express or highly express the epidermal growth factor receptor (EGFR). In lung cancer, deregulation of EGFR is seen mainly in NSCLC: EGFR is highly expressed in NSCLC at levels varying from 32–80% [4-6]. Abnormal signal transduction arising from the receptor is implicated in the growth and proliferation of these tumours. Thus, disruption of the EGFR signal transduction pathway is an ideal target for anticancer therapy.

Gefitinib is an orally active small molecule EGFR tyrosine kinase inhibitor (EGFR-TKI). The tolerability and efficacy of gefitinib have been explored extensively in the West and Japan. In a Phase I trial, Nakagawa et al compared the safety profile, pharmacokinetic parameters and antitumour activity of gefitinib in patients with solid malignant tumours in Japan, the USA and Europe, and no significant difference was found for ethnicity [7]. However, severe interstitial pneumonia related to gefitinib therapy was more common in the Japanese population [8,9]. In IDEAL ('Iressa' Dose Evaluation in Advanced Lung cancer) 1, which included 102 Japanese patients with advanced NSCLC, the response rate of Japanese patients taking gefitinib was significantly higher than that of non-Japanese patients but bias of baseline factors between strata, not ethnicity, was thought to account for the difference [10]. But Owing to the paucity of current data on gefitinib between defferent races, the possibility of differences in the toxicity and efficacy of gefitinib for ethnicity cannot be excluded. At present, there are few data regarding the tolerability and efficacy of gefitinib in Chinese patients.

Patients with advanced NSCLC who had no alternative therapeutic options have been able to receive gefitinib treatment in a worldwide compassionate-use programme. Here we report the outcome of treatment with oral gefitinib 250 mg/day in Chinese patients with advanced NSCLC participating in the compassionate-use programme at Peking Union Medical College Hospital.

## Methods

### Patient population

Patients aged ≥18 years with histologically or cytologically confirmed advanced or metastatic NSCLC were eligible for enrolment into the compassionate-use programme after providing written, informed consent. They were required to have failed prior chemotherapy, and had no other treatment options available. Patients who had received chemotherapy were included if the treatment ended >28 days prior to the study. Other eligibility criteria included: adequate bone marrow function (white blood cell count >4 × 10^9^/L, absolute neutrophil count >1.5 × 10^9 ^cells/L, platelet count >100 × 10^9^/L); liver function (total bilirubin <34 μmol/L, aspartate aminotransferase [AST] <40 IU/L, alkanine aminotransferase [ALT] <41 IU/L); renal function (serum creatinine <150 μmol/L, blood urea nitrogen <10.7 mmol/L); PaO_2 _>60 mmHg.

Exclusion criteria included a serious pre-existing medical condition (eg uncontrolled infection, interstitial pneumonia or pulmonary fibrosis, severe chronic diarrhoea), pregnancy and lactation.

### Drug administration

One oral gefitinib tablet (250 mg) was taken at about the same time each day. Gefitinib was administered every day without interruption unless disease progression or unacceptable toxicity occurred.

### Evaluation before and during treatment

In this study, 28 days of treatment was defined as one treatment cycle. Baseline evaluation included medical history and physical examination, electrocardiogram, chest X-ray, thorax computed-tomography scan and ultrasonography of the upper abdomen. Laboratory investigations included complete blood counts, urinalysis, and renal and liver function tests. Performance status (PS) was evaluated according to Eastern Cooperative Oncology Group (ECOG) criteria. Brain magnetic resonance imaging and radionuclide bone scans were only performed if metastatic disease was suspected according to the clinical manifestations of each patient. Regarding interstitial lung disease, we paid attention to clinical respiratory symptoms (e.g. dyspnea, cough) and radiographic findings of patients, and monitored PaO_2 _during therapy. Patients were evaluated after the first and third cycles of therapy, then every three cycles. Tumour response was evaluated according to Response Evaluation Criteria In Solid Tumors [11]. In line with the criteria, all patients included in the study were assessed for response to treatment. Each patient was assigned to one of the following categories: complete response (CR); partial response (PR); stable disease (SD); progressive disease (PD); early death from lung cancer; early death from toxicity; early death because of other disease; unknown (not assessable or insufficient data). All AEs were recorded and graded according to National Cancer Institute Common Toxicity Criteria version 2.0.

### Assessment of QoL

QoL was assessed using the Chinese version of the European Organization for Research and Treatment of Cancer (EORTC) core questionnaire, the Quality of Life Questionnaire (QLQ)-C30 (version 3.0) and the supplemental lung-cancer-specific module QLQ-LC13 [12]. Following the scoring procedure recommended by the EORTC, scores were converted into linear transformation ranging from 0–100. For the functional and global health status/QoL, higher scales represent better functioning. For symptoms, a higher score represents worse symptoms [13]. QoL and disease-related symptoms were assessed before the start of the therapy and then at the end of each cycle in the first 3 months of treatment.

### Statistical analysis

Logistical regression test models were used to identify baseline factors (gender, PS, histology, TNM stage and prior chemotherapy) that might independently predict tumour response. Median progression-free survival (PFS) and overall survival (OS) were calculated using the Kaplan-Meier method and a log-rank test was used to detect differences OS between strata.

Changes in symptoms and QoL were assessed in two different ways. Firstly, the mean scores of QoL and disease-related symptoms at baseline were compared with those at the end of the second cycle of treatment using a paired-sample t test. Secondly, the response rates of symptoms and QoL were calculated. For the assessment of symptoms and QoL, the classification system suggested by Stephens et al was used [14]. The results were presented as response (ie improvement, control or prevention), no response and nonevaluable cases in the form of percentages. The classification system [15,16] has been used successfully to evaluate QoL and symptoms in patients with lung cancer. Response rates for QoL and disease-related symptoms were compared between patients with and without objective tumour response using Pearson's χ^2 ^test or Fisher's exact test.

## Results

### Patient characteristics

Thirty-one eligible patients were enrolled into the EAP at Peking Union Medical College Hospital between October 2002 and October 2003. Patient characteristics are listed in Table 1. The patient series included 18 (58%) men and 13 (42%) women between 28 and 85 years of age (median age 64). All patients had received at least one platinum-based regimen and most had received more than two regimens (different combinations including platinum, taxane, docetaxel and gemcitabine). Ten (32.3%) patients had squamous-cell carcinoma (SCC) and 20 (64.5%) had adenocarcinoma. Most patients (61.3%) had an ECOG PS of 0–1, 25.8% had a PS of 2 and 12.9% had a PS of 3. TNM stages were as follows: stage IIIa, 1 patient; stage IIIb, 4 patients; stage IV, 26 patients.

### Toxicity

All patients were assessed for toxicity. Twenty-three (74%) patients had at least one AE. Treatment-related toxicities are listed in Table 2. Almost all AEs (but for one grade 3 acneform rash) were mild (grade 1 or 2). The most frequently reported AEs during treatment were acneform rash (67.7%) and diarrhoea (35.5%). Four (12.9%) patients had grade 1 or 2 stomatitis. Other AEs included nausea (6.5%), vomiting (3.2%), increased ALT (3.2%) and increased AST (3.2%). The majority of AEs were transient and reversible. Only one patient withdrew from treatment due to an AE (on the 50th day of treatment with grade 3 skin rash and exacerbation of dysphagia). This patient had SCC, a PS of 3 and mediastinal lymph node metastases before treatment. The patient suffered moderate haemoptysis prior to gefitinib therapy, which reduced to a mild severity during therapy. Fifteen days after withdrawal from treatment, the patient died of massive haemoptysis.

### Patient response

The assessment of patient response is listed in Table 3. Of the 31 patients, 1 (3.2%) achieved CR and 10 (32.3%) achieved PR with an overall objective response rate of 35.5% (95% confidence interval [CI]: 18.6–52.3). SD was documented in 7 (22.6%) patients and the overall DCR (CR+PR+SD) was 58.1% (95% CI: 49.2–67.0). The response rate of adenocarcinoma was significantly higher than that of squamose carcinoma [50% (10/20) vs. 10% (1/10)]. Multivariate logistic analysis also showed that the odds of objective response were 10 times higher (odds ratio 10; 95% CI: 1.0 to 93.4; p = 0.028) for patients with adenocarcinoma than for patients with other tumour histologies. Of the 31 patients who received treatment, 2 died due to PD before efficacy assessment. Both these patients had SCC and a PS of 3, and no improvement in either patient had been observed during gefitinib treatment.

Median PFS was 5.5 months (95% CI, 1.6 to 9.4); median OS was 11.5 months (95% CI, 5.6 to 17.3). The tumor response was associated with improved OS. Median OS of patients with response was significantly higher than that of those with no response (log-rank test p = 0.0058) (17 *vs. *4.4 months).

### QoL and symptom improvement

At baseline, 25 (81% [25/31]) patients returned QoL questionnaires. All 25 patients returned the questionnaires at the first cycle of therapy, 23 (93% [23/25] of those patients returning questionnaires and remaining alive) at the second cycle of therapy, and 18 (74% [18/24] of those patinets returning questionnaires and remaining alive) at the third cycle. The most frequently reported general symptoms were fatigue (100%) and appetite loss (68%), while dyspnoea (100%) and coughing (84%) were the most frequently reported respiratory symptoms. The changes in the mean scores for symptom scales, functioning and global QoL scales are presented in Table 4. A statistically significant increase in mean score was observed for physical functioning, role functioning, emotional functioning, social functioning and global QoL after 8 weeks of treatment. There was also a trend towards a higher score (p = 0.08) for cognitive functioning. Mean scores for two general symptoms (fatigue and appetite loss) and disease-related symptoms (dyspnoea, coughing, pain in chest, pain in arm or shoulder and pain in other parts) decreased statistically.

**Figure 1:**
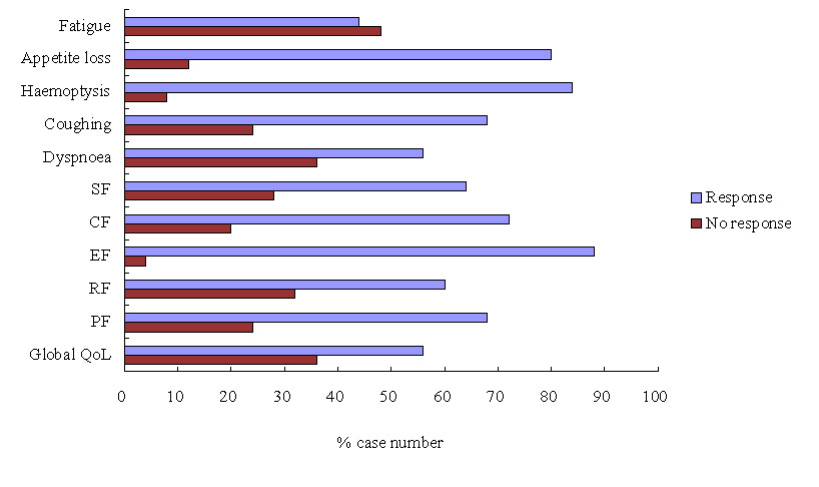
Patient response for QoL and main symptoms QoL, quality of life; PF, physical functioning; RF, role functioning; EF, emotional functioning; CF, cognitive functioning; SF, social functioning

Patient responses for functioning, global QoL and main symptoms are shown in figure 1. A >50% response rate was observed in all five functioning criteria and global QoL. With regard to QoL, the highest response rate was observed for emotional functioning (88%), followed by cognitive (72%), physical (68%), social (64%) and role functioning (60%), and global QoL (56%).

Haemoptysis had the highest response rate (84%), the rate of improvement (63%) being just lower than that of appetite loss among the five symptoms. The response rates for dyspnoea and coughing were 56% and 68%, respectively. For fatigue and appetite, the response rates were 44% and 80%, respectively.

Differences in response rates for symptoms and QoL between patients with or without objective tumour response are shown in Table 5. Response rates of physical functioning, role functioning, social functioning and global QoL were significantly higher in patients with objective tumour response than in those without. Response rates for emotional functioning and cognitive functioning were also higher in patients with tumour response, though the difference was not statistically significant. Differences were also observed in response rates for symptoms. For dyspnoea, coughing, appetite loss and fatigue, the response rates in the objective responders were higher than those in nonresponders. No significant association was found between haemoptysis and objective tumour response.

## Discussion

A daily oral 250 mg dose of gefitinib was well tolerated in this series of Chinese patients with advanced NSCLC, who received gefitinib as part of a compassionate-use programme. AEs were mild and reversible and different to those associated with conventional chemotherapy, such as neutropenia, thrombocytopenia and neuropathy. No haematological or neural toxicity was observed in the study. As with prior clinical trials evaluating the toxicity of gefitinib, the most frequently reported AEs were acneform rash and diarrhoea (grade 1 or 2). Four patients developed grade 1 or 2 stomatitis during treatment, which has previously been associated with gefitinib treatment in 7.8% of patients in IDEAL 1 but was not reported in IDEAL 2 [17].

Severe acute interstitial pneumonia is the most serious AE that has been linked with gefitinib therapy. Inoue et al recently reported that 4 of 18 patients receiving gefitinib in their clinic developed severe acute interstitial pneumonia [8]. The authors stated that 291 of 17,500 patients (1.7%) treated with gefitinib in Japan had developed suspected interstitial pneumonia or acute lung injury. In contrast to the higher incidence of interstitial pneumonia, this severe AE was lower in the rest of the world [9]. The worldwide frequency of interstitial lung disease to date in ~92,750 patients who have received gefitinib is <1.0% [18]. The ethnicity between Chinese and Japanese people is probably similar, but we saw no evidence of interstitial pneumonia in the series of patients. Although no patients developed acute interstitial pneumonia, the possibility of drug-related interstitial lung disease could not be excluded because of the small number of patients in our series.

The response rate (35.5%) was higher than that of recently presented Phase II trials (18.4% and 11.8% in patients receiving gefitinib 250 mg/day in IDEAL 1 and 2, respectively). The DCRs were 54.4% and 42% for the 250 mg/day dose in IDEAL 1 and 2, respectively, in contrast to 58.1% in our study. Unlike IDEAL 2, which was performed in the USA, IDEAL 1 was a global trial that recruited patients from 43 centres across Europe, Australia, South Africa and Japan and included a total of 102 Japanese patients with advanced NSCLC. The response rate for Japanese patients was higher than that of non-Japanese patients (27.5% versus 10.45%; odds ratio 3.72; p = 0.0023). However, the difference in response rate for ethnicity was not verified using multivariate logistical regression analysis. The difference in response rate between Japanese and non-Japanese patients was attributed to bias of baseline predictive factors of patients (gender, PS and histology) [19,10].

QoL is an important endpoint for assessment of gefitinib treatment and has been included in some phase I and phase II trials. In these trials, the Functional Assessment of Cancer Therapy – Lung questionnaire was used to assess QoL and symptoms. Its validation and sensitivity were verified. Significant improvements in symptoms and QoL were observed in IDEAL 1 and 2. In IDEAL 1, symptom and QoL improvement with a dose of 250 mg/day were 40.3% and 23.9%, respectively, and in IDEAL 2 they were 43.1% and 34.3%, respectively. In both trials, improvements in symptoms and QoL correlated well with tumour response [20].

In our patient series, the EORTC QLQ-C30 and its supplement module QLQ-LC13 were used for assessment of QoL and symptoms. Together, they are thought to be one of the best developed instruments for lung cancer assessment [21]. The questionnaires have been translated into >20 languages and validation of the standard Chinese version has been verified [22]. In our series of patients, mean scores of four functioning scales (physical, role, emotional and social functioning) and global QoL increased significantly after 8 weeks of treatment and were associated with high response from 56–88%. Mean score of cognitive functioning also increased, from 66 at baseline to 76 at the end of second cycle with response of 72% (not statistically significant; p = 0.08). General and disease-related symptom scores increased from 44% to 84%. Importantly, improvement of symptoms and QoL correlated with objective tumour response. Patients with objective tumour response had higher rates of symptom and QoL response. The results indicate that placebo effect is unlikely to explain completely the improvement in symptoms and QoL with gefitinib, though there is no placebo control in our patient series or the EAP. Emotional functioning response was observed in all patients with objective tumour response, but this was not statistically significant when compared with nonresponders (100% versus 79%; p = 0.23). As emotional functioning contained more subjective questions, such as "Did you feel tense?", "Did you worry?", "Did you feel irritable?" and "Did you feel depressed?", the limited difference of emotional functioning response between responders and nonresponders with gefitinib cannot completely exclude the possibility of placebo effect. Thus, a placebo control group should be included in future studies of QoL and symptom improvement with gefitinib.

## Conclusions

In conclusion, the data from this patient series show that an oral dose of gefitinib 250 mg/day is well tolerated and has significant antitumour activity in Chinese patients with advanced NSCLC who had failed previous chemotherapy and for whom no other treatment options were available. Although clinical trials are needed to confirm these positive findings, the data suggest that treatment with gefitinib may be beneficial for some Chinese patients who do not respond to chemotherapy and have poor prognosis.

'Iressa' is a trademark of the AstraZeneca group of companies

## Competing interests

None declared.

## Authors' contributions

XLM and LYL designed the experiments and wrote the manuscript. XTZ, SLW and MZW participated in patient's follow-up and tumour response evaluation.

## Pre-publication history

The pre-publication history for this paper can be accessed here:



## Figures and Tables

**Table 1:** Characteristics of patient series (n = 31)

Median age, years (range)	64 (28–85)
Gender, n (%)	
male	18 (58)
female	13 (42)
ECOG PS, n (%)	
0	3 (9.7)
1	16 (51.6)
2	8 (25.8)
3	4 (12.9)
Histology, n (%)	
squamous	10 (32.3)
adenocarcinoma	20 (64.5)
unspecified	1 (3.2)
TNM staging, n (%)	
IIIa	1 (3.2)
IIIb	4 (12.9)
IV	26 (83.9)
Prior chemotherapy, n (%)	
1 regimen	5 (16.1)
2 regimens	10 (32.3)
≥3 regimens	16 (51.6)

ECOG, Eastern Cooperative Oncology Group; PS, performance status

**Table 2:** Treatment-related toxicity (n = 31)

**Adverse events**	**Grade**						**Total**	
			
	**1**		**2**		**3**			
	
	**n**	**%**	**n**	**%**	**n**	**%**	**n**	**%**
Acneform rash	17	54.8	3	9.7	1	3.2	21	67.7
Diarrhoea	10	32.3	1	3.2	-	-	11	35.5
Nausea	1	3.2	1	3.2	-	-	2	6.5
Vomiting	-	-	1	3.2	-	-	1	3.2
Stomatitis	4	12.9	-	-	-	-	4	12.9
Increased ALT	1	3.2	-	-	-	-	1	3.2
Increased AST	1	3.2	-	-	-	-	1	3.2

ALT, alkaline aminotransferase; AST, aspartate aminotransferase

**Table 3:** Tumour response (n = 31)

	**Patients**
Complete response, n (%)	1 (3.2)
Partial response, n (%)	10 (32.3)
Stable disease, n (%)	7 (22.6)
Progressive disease, n (%)	11 (35.5)
Early death, n (%)	2 (6.5)
Response rate, % (95% CI)	35.5 (18.6–52.3)
Disease control rate, % (95% CI)	58.1 (49.2–67.0)

CI, confidence interval

**Table 4:** Changes in mean score for QoL and symptoms

**QoL items**	**Baseline mean (standard deviation)**	**2nd cycle mean (standard deviation)**	**p value**
Global QoL	36.0 (24.7)	55.4 (16.2)	0.01
Physical functioning	47.47 (24.74)	65.22 (20.59)	0.01
Role functioning	42.67 (35.05)	56.52 (25.99)	0.03
Emotional functioning	67.67 (28.39)	84.78 (19.57)	<0.01
Cognitive functioning	66.00 (28.66)	76.09 (18.69)	0.08
Social functioning	42.67 (32.30)	60.87 (26.88)	0.01
Symptoms items			
Fatigue	64.89 (25.99)	45.89 (22.30)	<0.01
Appetite loss	41.33 (36.36)	20.29 (24.08)	0.01
Dyspnoea	60.44 (28.16)	39.61 (23.88)	<0.01
Coughing	58.67 (36.36)	28.99 (30.66)	<0.01
Haemoptysis	12.00 (18.95)	7.25 (17.28)	0.21
Pain in chest	36.00 (33.22)	17.39 (19.77)	0.04
Pain in arm or shoulder	24.00 (28.09)	13.04 (19.43)	0.03
Pain in other parts	25.33 (27.69)	14.49 (22.08)	0.02

QoL, quality of life

**Table 5:** QoL and symptom response rates among objective tumour responders and nonresponders

**Items**	**Responders (n = 11)**		**Nonresponders (n = 14)**		**p value**
	
	**n**	**%**	**n**	**%**	
QoL					
Global QoL	10	91	4	29	0.004
PF	10	91	7	50	0.042
RF	10	91	5	36	0.012
EF	11	100	11	79	0.23
CF	10	91	8	57	0.09
SF	10	91	6	43	0.033
Symptoms					
dyspnoea	9	82	5	36	0.021
coughing	10	91	7	50	0.042
haemoptysis	11	100	10	71	0.105
appetite loss	11	100	9	64	0.046
fatigue	8	73	3	21	0.01

QoL, quality of life; PF, physical functioning; RF, role functioning; EF, emotional functioning; CF, cognitive functioning; SF, social functioning
